# Immunohistochemical and other prognostic factors in B cell non Hodgkin lymphoma patients, Kampala, Uganda

**DOI:** 10.1186/1472-6890-9-11

**Published:** 2009-12-16

**Authors:** Lynnette K Tumwine, Claudio Agostinelli, Cristina Campidelli, Emmanuel Othieno, Henry Wabinga, Simona Righi, Brunangelo Falini, Pier Paolo Piccaluga, Wilson Byarugaba, Stefano A Pileri

**Affiliations:** 1 Department of Pathology, Makerere University, College of Health Sciences, PO Box 7072, Kampala, Uganda; 2 Unit of Hematopathology, Department of Haematology and Oncological Sciences "L and A Seràgnoli"/Interdepartmental Centre for Cancer Research "G Prodi", Bologna University School of Medicine, Bologna, Italy; 3 Laboratory of Haematopathology, Institute of Haematology, Perugia University School of Medicine, Perugia, Italy

## Abstract

**Background:**

Non Hodgkin lymphomas are the most common lymphomas in Uganda. Recent studies from developed countries have shown differences in survival for the different immunophenotypes. Such studies are lacking in Africa where diagnosis is largely dependent on morphology alone. We report immunohistochemical and other prognostic factors in B cell non Hodgkin lymphoma patients in Kampala, Uganda.

**Methods:**

Non Hodgkin lymphoma tissue blocks from the archives of the Department of Pathology, Makerere University College of Health Sciences, Kampala, Uganda, from 1991-2000, were sub typed using haematoxylin and eosin, Giemsa as well as immunohistochemistry. Using tissue micro array, 119 biopsies were subjected to: CD3, CD5, CD10, CD20, CD23, CD30, CD38, CD79a, CD138, Bcl-6, Bcl-2, IRTA-1, MUM1/IRF4, Bcl-1/cyclin D1, TdT, ALKc, and Ki-67/Mib1. Case notes were retrieved for: disease stage, chemotherapy courses received and retrospective follow up was done for survival.

**Results:**

Non Hodgkin B cell lymphomas comprised of Burkitt lymphoma [BL] (95/119) diffuse large B cell lymphoma (19/119), mantle cell lymphoma (4/119) and precursor B lymphoblastic lymphoma (1/119).

For Burkitt lymphoma, good prognosis was associated with receiving chemotherapy, female gender and CD30 positivity. Only receiving chemotherapy remained significant after Cox regression analysis. Diffuse large B cell lymphomas with activated germinal centre B cell (GCB) pattern (CD10+/-, BCL-6+/-, MUM+/-, CD138+/-) had better survival (98.4 months; 95% CI 89.5 -107.3) than the others (57.3 months; 95% CI 35.5 - 79.0) p = 0.027 (log rank test).

**Conclusions:**

Activated GCB diffuse large B cell lymphoma had a better prognosis than the others. For Burkitt lymphoma, not receiving chemotherapy carried a poor prognosis. Availability of chemotherapy in this resource limited setting is critical for survival of lymphoma patients.

## Background

Non Hodgkin B cell lymphomas are heterogeneous in morphology, immunophenotype and response to therapy. Recent studies have shown differences in survival based on their molecular profile[1].

In developing countries, clinically aggressive subtypes such as Burkitt and diffuse large B cell lymphoma predominate and, unfortunately, result in poor outcome[2]. Factors that influence survival in non Hodgkin lymphomas in resource poor settings include socio economic status, stage of disease at presentation and getting a full course of treatment. In Uganda, several studies have described clinical factors associated with outcome of Burkitt lymphoma [3,4].

In the developed countries, several methods including gene profiling and immunohistochemistry have been used for predicting prognosis [5,6]. Using the gene expression profile of germinal centre B and activated B cell, diffuse large B cell lymphoma (DLBCL) was subdivided into 3 prognostic groups. However, there are several draw backs of gene expression profiling especially in resource constrained countries such as Uganda. It requires the use of optimally cryopreserved or fresh tissues as well as DNA micro array technology which is more costly than immunohistochemistry on paraffin sections.

Recently, several workers have used germinal centre and activated B cell immunohistochemical markers on paraffin embedded tissue blocks to classify DLBCL into three prognostic groups[7,8].

These include: (a) activated non GCB (CD10-, Bcl-6-, MUM1/IRF4 ±, CD138+); (b)activated GCB (CD10+, Bcl-6+, MUM1/IRF4 ±, CD138+); and (c) non activated GCB (CD10+, Bcl-6+, MUM1/IRF4-, CD138-). They showed that patients with a germinal centre B cell profile have a much better prognosis than those with the activated B cell type. Such studies have hitherto not been carried out in Uganda. We report immunohistochemical and other prognostic factors in B cell non Hodgkin lymphoma patients in Kampala, Uganda

## Methods

### Study design and sampling

A cross sectional descriptive design was used for lymphoma diagnosis and immunophenotyping, while a retrospective cohort was used to determine survival. For the cross sectional study, haematoxylin and eosin and Giemsa staining was carried out in the Department of Pathology, Makerere University and immunohistochemistry in the Unit of Hematopathology, Institute of Hematology and Clinical Oncology "L. & A. Seràgnoli", Bologna University School of Medicine, Bologna, Italy. One hundred and twenty nine patients' biopsies diagnosed between 1991-2000 as non Hodgkin lymphoma were sub typed using tissue microarray (TMA) and immunohistochemistry with CD3, CD5, CD10, CD20, CD23, CD30, CD38, CD79a, CD138, Bcl-6, Bcl-2, IRTA-1, MUM1/IRF4, Bcl-1/cyclin D1, TdT, ALKc, and Ki-67/Mib1.

For the retrospective cohort study we retrieved patients' case notes from the Uganda Cancer Institute in order to obtain details of the patients' disease stage, type of chemotherapy, number of courses received, whether dead or alive, time to death. Cancer registry data was also used when the addresses of the patients fell within Kyadondo County, the area covered by the Kampala Cancer Registry. One of us (LKT) and two research assistants followed up patients whose survival status was not clear. The follow up involved tracing patients to their homes (district, sub-county, parish and village) in the different regions of Uganda.

The patients had been treated at the Uganda Cancer Institute which is the oldest unit for cancer treatment in the country. It began as a centre for the treatment of Burkitt lymphoma patients in the 1960s and has two units: the solid tumor treatment unit and the lymphoma treatment centre. Those with Burkitt lymphoma received COM[9] (cyclophosphamide, vincristine, intrathecal methotrexate) whereas those with other non Hodgkin lymphomas received CHOP (cyclophosphamide, adriamycin, vincristine and prednisolone).

### Tissue micro array construction (TMA)

Haematoxylin and eosin (H&E) stained slides were used to identify the representative tumor fields that were marked and correspondingly identified on the tissue blocks.

Tissue cylinders of diameter of 1 mm were punched from the marked areas on each block and incorporated into a recipient paraffin block using a precision instrument, the tissue arrayer (Beecher Instruments, Silver Spring, Maryland, USA). For adequate sampling each specimen was represented in duplicate using 1 mm cores in the recipient block. Three TMA recipient blocks were made; two of these had 48 punches each of Burkitt lymphoma while the other one had 33 punches of other non Hodgkin lymphomas.

### Immunohistochemistry

Four-μm thick sections were cut from TMAs, coated on electrically charged slides, re-hydrated, and submitted to antigen retrieval in ethylene diamine tetra acetic acid (EDTA) 1 mM (pH 8.0) by micro-waving twice for 5 minutes at either 750 or 900 W, that was very efficient according to previous experience[10].

After cooling, the slides were put on a TechMate 500 immunostainer and incubated for 30 minutes at room temperature with antibodies against CD3, CD5, CD10, CD20, CD23, CD30, CD38, CD79a, CD138, Bcl-6, Bcl-2, IRTA-1, MUM1/IRF4, Bcl-1/cyclin D1, TdT, ALKc, and Ki-67/Mib1. Details on the antibodies, sources, dilutions and antigen retrieval are listed in Table 1.

**Table 1:** Primary antibodies used for the study.

Antibody	Clone	Source	Antigen retrieval	Dilution
CD3	SP7	Immunotech	EDTA 750 W	1:250

CD5	54/F6	Dako	EDTA 900 W	1:10

CD10	56C6	Novocastra	EDTA 900 W	1:5

CD20	L26	Dako	EDTA 750 W	1:200

CD23	1B12	Novocastra	EDTA 900 W	1:30

CD30	Ber- H2	Prof. Falini *	EDTA 900 W	1:3

CD38	SPC32	Novocastra	EDTA 750 W	1:10

CD79a	JCB117	Prof. Mason §	EDTA 750 W	1:10

CD138	-	Neomarkers	EDTA 900 W	1:20

BCL-1	SP4	Neomarkers	EDTA 900 W	1:20

BCL-6	PG-B6p	Prof. Falini *	EDTA 900 W	Undiluted

BCL-2	124	Prof. Mason §	EDTA 900 W	1:3

IRTA-1	Mum2EC	Prof. Falini *	EDTA 900 W	1:2

MUM1/IRF4	-	Prof. Falini *	EDTA 900 W	1:2

TdT	-	Dako	None	1:30

ALKc	-	Prof. Falini *	EDTA 900 W	1:2

Ki-67	Mib-1	Dako	EDTA 900 W	1:20

* Kindly provided by Prof. Brunangelo Falini, Perugia University, Perugia, Italy.

§Kindly provided by Prof. David Y. Mason, Oxford University, Oxford, before his premature death.

The antibodies were detected by either the alkaline phosphatase anti-alkaline phosphatase immunocomplexes (APAAP) technique or the Envision^+ ^technique [11].

### Data management, analysis and statistical issues

For the descriptive cross sectional study, a sample size of 112 was calculated using a formula by Kish and Leslie[12]. In this calculation we assumed that the prevalence of B cell non Hodgkin's lymphoma in the total population of non-Hodgkin lymphoma was 92.1% according to a Ugandan study[2] with a precision of 5% and 95% confidence interval.

For the retrospective cohort design, a sample size of 52 was calculated using a formula by Fleiss with 80% power and 95% confidence interval [12]. We assumed the expected outcome among patients with NHL of germinal centre origin would be 86% and the expected outcome among patients with NHL of non germinal centre origin would be 63%.

Data was collected and entered into the computer using EPI INFO software (supplied by CDC and WHO) for storage and initial analysis. Further analysis was done using SPSS software. For continuous variables, the relevant measures of central tendency (means for normally distributed data and medians and inter-quartile ranges for skewed data) were used to explore the data.

Kaplan-Meier curves and the log rank test were used to determine survival. To determine factors associated with overall survival, univariate and multivariate Cox hazards regression analysis was carried out. A p value of less than 0.05 was considered significant.

### Ethical issues

Permission to carry out the study was obtained from the Makerere University Faculty of Medicine Research and Ethics Committee.

### Study limitations

This was a retrospective rather than prospective study making it difficult to get good socio-demographic information. Clinical outcome predictors such as the International prognostic Index were not complete. Lactate dehydrogenase was not routinely done in the patients.

## Results

### Background characteristics

The median age was 9.0 (inter-quartile range 6- 15.5) years. The youngest patient was 2 years and the oldest was 64 years. The mean age was 15.7 (SD 15.5) years.

As expected, 79.8% of the patients had Burkitt lymphoma, followed by diffuse large B cell lymphoma (16.0%), mantle cell lymphoma (3.4%), and precursor B lymphoblastic lymphoma (0.8%) (Table 2)

**Table 2:** Distribution of B cell non Hodgkin lymphoma by diagnosis and immunophenotype

Diagnosis				
Marker	Burkitt lymphoma (%positive)	Diffuse large B cell lymphoma (% positive)	Precursor B lymphoblastic lymphoma	Mantle cell lymphoma
CD10	74/79(93.7)	3/18 (16.7)	1/1	0/4
CD20	95/95(100)	19/19(100)	1/1	4/4
BCL6	46/69(66.7)	2/19(10.5)	0/1	1/4
CD79a	48/68(70.6)	6/16(37.5)	1/1	2/4
CD38	35/68(51.5)	3/16(18.8)	0/1	0/4
MUM-1/IRF4	1/77(1.3)	4/18(22.2)	0/1	0/4
EBER	79/86(91.9)	6/17(35.3)	1/1	0/4
Tdt	0/95(0)	0/19(0)	1/1	0/4
BCL2	6/79(7.6)	7/19(36.8)	1/1	4/4
CD3	0/86(0)	0/19(0)	0/1	0/3
CD138	43/95(45.3)	10/15(66.7)	0/1	4/4
CD30	35/95(36.8)	2/17(11.8)	0/1	0/4

### Burkitt lymphoma survival

The male patients had a mean survival of 3.3 (95% CI 0.81-5.8) months while the females had a mean survival of 17.7 (95% CI 8.3-27.0) months, p = 0.028. The CD30 negative patients had a mean survival of 6.8 (95% CI 0.0 - 14.7) while the CD30 positive had a mean survival of 12.3 (95% CI 7.3 - 17.3), p = 0.0169.

Only receiving chemotherapy remained significant after Cox regression analysis (Table 3)

**Table 3:** Results of Cox regression analysis for survival, Burkitt lymphoma cases, Uganda

Variable	Hazard's ratio (95.0% CI)	p value
Received chemotherapy	0.15 (0.001-0.195)	0.001

CD30	0.427 (0.098-1.863)	0.257

Male	4.207 (0.775-22.84)	0.096

CI Confidence Intervals

### Burkitt lymphoma survival by EBER status

Overall 97.3% were EBER positive. Survival information was available on 25 patients whose EBER results were available. Of these, 24 (96%) were EBER positive. The mean survival for the 24 was 9.28(95%CI 3.27-15.29 months). However there was only one EBER negative patient whose survival was 1.2 months.

### Burkitt lymphoma: EBER status and CD30 positivity

A total of 55 cases had both CD30 and EBER results. Of the 24 CD30 positive, 23(95.8%) were EBER positive. Of the 31 CD30 negative, 30(96.8%) were EBER positive. The difference was not statistically significant (p = 0.83); and because of this, the EBER result was not entered into Cox regression analysis.

### Diffuse large B cell lymphoma survival

**Figure 1:**
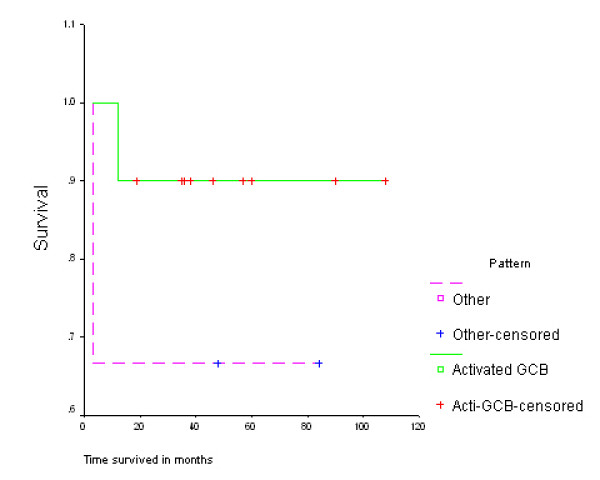
**Kaplan Meier survival curve for patients with Diffuse Large B Cell Lymphoma, Kampala, Uganda**.

Diffuse large B cell lymphomas with activated germinal centre B cell (GCB) pattern (CD10+/-, BCL-6+/-, MUM+/-, CD138+/-) had better survival (98.4 months; 95% CI 89.5 -107.3) than the others (57.3 months; 95% CI 35.5 - 79.0) p = 0.027 (log rank test) (Figure 1)

## Discussion

The aim of this paper was to classify B cell non Hodgkin lymphomas in Uganda using immunohistochemical markers and correlate this and other factors to patient outcome.

In the current study, B cell non Hodgkin lymphomas seem to be affecting mainly young people, with a median age of 9 years. The mean age was 15.7 years consistent with results of previous similar studies from Uganda [2], but much lower than that reported from Kenya by Cool and Bitter [13].

### Overall survival of patients with B cell non Hodgkin lymphomas

#### Burkitt lymphoma

The overall survival of patients with B cell non Hodgkin lymphomas was 60 months but only 12.7 months in patients with Burkitt lymphoma. This survival of patients with Burkitt lymphoma in this series is surprisingly much lower than what was reported from the same centre in the 1970s and 1980s by Olweny and others [14]. This difference could possibly be due to the fact that Olweny's studies were done on a cohort that was meticulously followed up, unlike the current investigation in which we studied routine patients who came to the Uganda Cancer Institute for treatment and who were not particularly followed up or sought after to complete treatment [14].

In the current study factors associated with better survival included receiving chemotherapy, female gender and CD30 positivity.

As expected those who did not receive chemotherapy died within the first month after presentation to hospital. The possible reason for this is that the Uganda Cancer institute is grossly under funded and therefore patients have to buy anticancer drugs from private pharmacies. Unfortunately, most patients are poor and therefore cannot afford the drugs[15]. Many patients are referred from rural areas to the main cancer treatment centre in Kampala at the Uganda Cancer Institute and this constitutes further delay.

In Uganda, the standard regimen for treating Burkitt lymphoma is cyclophosphamide, vincristine, prednisolone (COM) and intrathecal methotrexate for central nervous system disease prophylaxis [9,14]. In our study, those who received chemotherapy with COM had a better overall survival than those who did not. Endemic Burkitt lymphoma is characterised by a very high proliferative index nearing 100%, and the disease is very rapidly progressive with a doubling time of 24 hours and is fatal if not treated early with intensive chemotherapy regimens [16].

Another factor associated with poor survival was male gender. Similar observations have been made by other authors in studies on childhood cancers [17,18]. The reasons for the difference in prognosis between males and females among patients with Burkitt lymphoma are not very clear. However, one explanation is that males have an inherent tendency to have higher rates of cell division than females [19]. The fact that the growth rate of the male embryo is higher than that of the female [20] has been suggested as lending credence to this hypothesis [21].

Of interest in our study was the high CD30 positivity (37%) in Burkitt lymphoma.

This is different from results of a previous study in the United Kingdom where CD30 positivity in Burkitt lymphoma patients was not so high: Jones and others found 18% (3/17) CD30 positivity in childhood Burkitt lymphoma in the West Midlands, UK [22,23].

In our study CD30 positive patients had a better survival than those who were CD30 negative. Previously, CD30 positivity has not been associated with good prognosis in Burkitt lymphoma [24]. Several authors have suggested that the CD30-CD30L (ligand) interaction may have a role in some non Hodgkin lymphomas including Burkitt lymphoma [23,25]. Kanavaros et al 1992 found that many CD30 positive non Hodgkin lymphomas were EBV positive [25].

In our patients, CD30 positivity was found among those with both "typical morphology and plasmacytoid features." [2]. As we noted previously, this might be related to the "postulated complex pathogenesis of BL" in Africa [26]. Of interest is the multistep oncogenetic mechanism (proposed by Klein [27]) "in which there is de-regulation of MYC gene and subsequent development of a malignant clone." However, it appears that more studies are needed to shed more light on the specific role of CD30 in Burkitt lymphomas.

#### Survival of patients with DLBCL

In our study, diffuse large B cell lymphomas fell into three distinct groups with independent prognostic significance. These included: (a) non activated GCB, (b) activated GCB, and (c) activated non GCB. These are similar to the groups identified by researchers in the developed countries [7].

Whereas studies in the developed countries have found that non activated GCB had the best prognosis [7], in our study we have found that patients with activated germinal centre B- cell lymphoma had the best prognosis. The reasons for this difference are not clear and given the small numbers will have to be confirmed by larger studies. The difference could also be a reflection of yet unrecognized molecular heterogeneity in the tumors [28].

The overall survival of the patients with DLBCL in our study was lower than that reported from the developed countries. This could be related to the fact that in Uganda, patients with DLBCL are treated with cyclophosphamide, adriamycin, vincristine, prednisolone (CHOP) rather than the more effective cyclophosphamide, adriamycin, vincristine, prednisolone plus Rituximab (CHOP-R) that has become the standard of care in the developed countries [29].

## Conclusion

Immunohistochemistry on paraffin embedded tissue blocks using selected GCB and activation markers has yielded important information that predicts the outcome of patients with non Hodgkin B cell lymphomas in Uganda. Generally the Ugandan patients studied had a very poor prognosis. A number of factors including lack of timely chemotherapy seem to be responsible for this.

## Conflict of interests

The authors declare that they have no competing interests.

## Authors' contributions

LKT conceived the idea, collected the data, and wrote the manuscript. CA collected data CC collected data. EO contributed tissue blocks and collected data. HW contributed tissue blocks and survival data. SR did the immunohistochemistry and in situ hybridisation. WB reviewed and revised the manuscript. PPP reviewed and revised the manuscript. SAP reviewed and revised the manuscript. All authors read and approved the final version of the manuscript.

## Pre-publication history

The pre-publication history for this paper can be accessed here:

http://www.biomedcentral.com/1472-6890/9/11/prepub
